# Intestinal Endotoxins as Co-Factors of Liver Injury in Obstructive Jaundice

**DOI:** 10.1155/1996/75037

**Published:** 1996

**Authors:** B. Bülent Menteş, Ertan Tatlicioğlu, Gülen Akyol, Ömer Uluoğlu, Nedim Sultan, Erdal Yilmaz, Murat Çelebi, Ferit Taneri, Zafer Ferahköşe

**Affiliations:** 1 Department of Physiology Ankara University Medical School Ankara Turkey; 2 Departments of Surgery, Pathology Gazi University Medical School Ankara Turkey; 3 Departments of Microbiology Gazi University Medical School Ankara Turkey

## Abstract

The concept of endotoxin-mediated rather than direct liver injury in biliary obsruction was investigated using the experimental rat model of bile duct ligation (BDL) and small bowel bacterial overgrowth (SBBO). Small identical doses of intravenous endotoxin (bacterial LPS) caused a significantly more severe liver injury in rats with BDL, compared with sham-operated rats, suggesting the possible contribution of LPS in this type of liver damage. BDL was then combined with surgically created jejunal self-filling blind loops, which resulted in SBBO. Plasma LPS level increased significantly, and once again a more severe liver injury, determined by liver histology and serum gamma-glutamyl transpeptidase levels, was observed compared with the control group of rats with BDL+self-emptying blind loops. The data presented suggest that small amounts of exogenous LPS and/or the ordinarily innocous amounts of LPS constantly absorbed from the intestinal tract may be critical in the hepatic damage caused by obstruction of the biliary tract.


*HPB Surgery*, 1996, Vol. 9, pp. 61–69
Reprints available directly from the publisher
Photocopying permitted by license only

© 1996 OPA (Overseas Publishers Association)
Amsterdam B.V. Published in The Netherlands
by Harwood Academic Publishers GmbH
Printed in Malaysia

### Intestinal Endotoxins as Co-Factors of Liver Injury in Obstructive Jaundice

B. BÜLENT MENTEŞ, ERTAN TATLICIOĞLU, GÜLEN AKYOL,* ÖMER ULUOĞLU,* NEDIM SULTAN,** ERDAL YILMAZ, MURAT ÇELEBI,*** FERIT TANERI,* and ZAFER FERAHKÖŞE

*From the Departments of Surgery, Pathology, and **Microbiology, Gazi University Medical School and from the Department of Physiology, Ankara University Medical School, Ankara, Turkey

(*Received February 24, 1994*)

The concept of endotoxin-mediated rather than direct liver injury in biliary obsruction was investigated using the experimental rat model of bile duct ligation (BDL) and small bowel bacterial overgrowth (SBBO). Small identical doses of intravenous endotoxin (bacterial LPS) caused a significantly more severe liver injury in rats with BDL, compared with sham-operated rats, suggesting the possible contribution of LPS in this type of liver damage. BDL was then combined with surgically created jejunal self-filling blind loops, which resulted in SBBO. Plasma LPS level increased significantly, and once again a more severe liver injury, determined by liver histology and serum gamma-glutamyl transpeptidase levels, was observed compared with the control group of rats with BDL+self-emptying blind loops.The data presented suggest that small amounts of exogenous LPS and /or the ordinarily innocous amounts of LPS constantly absorbed from the intestinal tract may be critical in the hepatic damage caused by obstruction of the biliary tract.

KEY WORDS: Endotoxins; bile duct obstruction extrahepatic; liver injury

### INTRODUCTION

There is considerable evidence suggesting a relationship between endotoxins (bacterial lipopolysaccharides-LPS), cytokines, macrophage functions, and pathogenesis of liver injury in both acute and chronic liver diseases[1–4]. Whereas LPS may directly damage hepatocytes, a large number of LPS mediators originating from the monocyte/macrophage line are thought to work in concert with LPS at the cellular level and cause liver injury in defined circumstances[5–12]. The initial injury in a variety of liver injuries is to the Kupffer cells (KCs), and this injury impairs the ability of the liver to detoxify the ordinarily innocuous amounts of LPS, including those constantly absorbed from the gastrointestinal tract[13–15,1,3,4]. This KC depression brings about the consequent enhancement of endotoxic activity, and even minimal amounts of LPS might precipitate hepatic injury[1,4].

Extrahepatic bile duct obstruction and cholestasis have the well-known potential for liver injury, progression to cirrhosis and hepatocellular failure as evidenced by the study of the natural history of progressive cholestatic disorders[16–18]. Systemic endotoxemia is regularly found in those patients who have chronic extrahepatic bile duct obstruction[19,20]. Prolonged biliary obstruction is associated with a significant depression of the reticuloendothelial system (RES) phagocytic function and significantly higher mortality following endotoxin challenge[21]. The depressed function of the RES may allow spillover of even minute amounts of exogenous or gut-originated LPS into the circulation[22,23,4].

The concept of LPS as a key factor in liver injury has been studied in a number of experimental and clinical

*Address for correspondence:* B. Bülent Mentes, M.D. Naci Çakir Mah, 2. Sokak No:23/4 06450, Dikmen,Ankara, Turkey.

liver injuries[24,6,1,4]. In spite of the large body of knowledge about endotoxemia associated with obstructive jaundice, most of the studies have focused on the systemic effects of endotoxemia or extrahepatic manifestations such as renal failure[4,20–22].

In the present study, the effect of a small dose of exogenously administered LPS on liver injury was investigated, as the first step, in rats with extrahepatic bile duct obstruction. Extrahepatic bile duct obstruction was then combined with jejunal self-filling blind loops (SFBLs) in order to create small bowel bacterial overgrowth (SBBO). The resultant liver injury and plasma a LPS levels were evaluated and compared with appropriate controls in order to discuss the proposed complementary role of gut-originated endotoxins as key factors in liver injury caused by extrahepatic bile duct obstruction.

### METHODS

### Experimental Design and Surgical Procedures

Male Wistar rats, 12–20 weeks old and weighing 200–250 grams(g) were obtained from Gazi University Surgical Research Center. Animals were allocated to one of six groups:

Group 1($n$=6): Sham operation (sham for bile duct ligation).
Group 2($n$=8): Sham operation and 13 days later given intravenous (iv)LPS.
Group 3($n$=8): Bile duct ligation (BDL) for 14 days.
Group 4($n$=8): BDL for 14 days and iv LPS on the 13th day.
Group 5($n$=8): BDL + Jejunal self-filling blind loop (SFBL) for 14 days.
Group 6($n$=8): BDL + jujunal self-emptying blind loop (SEBL) for 14 days.

Rats were anesthetised with ketamine hydrochloride, 50 milligram (mg)/kilogram (kg) body weight intramuscularly (im) after an overnight fast. Operations were performed via an uppermidline incision under strict sterile conditions. BDL consisted of dissection of the supraduodenal portion of the common bile duct and division between 5/0 vycril ligatures. In the sham operation, the ligature was placed around the common bile duct and then removed.

In groups 5 and 6, BDL was combined with SFBL and SEBL, respectively. A modification of the method of Cameron *et al.*[25] was used and 10 centimeter (cm) jejunal SFBLs were created about 10 cm distal to the ligament of Treitz using a side-to-side anastomosis technique with continuous 8/0 polydiaxanone (PDS) sutures. This experimental model of SFBL has been previously shown to result in SBBO within one week following surgery[26,27]. The control group for this model consisted of rats with SEBLs, in which the corresponding jejunal segments were constructed using the same surgical techniques, but in an isoperistaltic fashion to empty aborally.

Postoperatively, all rats were kept in a temperature-controlled environment and allowed water and standard laboratory rodent chow ad libitum. 14 days later, all rats were again anaesthetised with ketamine hydrochloride im. Under strict sterile conditions, laparatomy was performed through the previous midline incision. A second set of sterile instruments was used for each animal after the skin was traversed with the first set. Peritoneal cultures were taken with a sterile applicator stick and cardiac blood was aspirated sterilely. Liver specimens from various sites were then obtained for histology. Last of all, luminal contents of jejunal loops were flushed with 10 milliliter (mL) saline and collected for anaerobic culture. (See below for technical details).

### *Endotoxin Challenge*

Rats of groups 2 and 4 were challenged with iv LPS on the 13th postoperative day, that is 24 hours before sacrifice. Lyophilized and gamma-irradiated LPS from E. coli 055:B5 (Sigma Chemical Co. St. Louis, Mo) was given through the tail vein following fresh reconstitution with pyrogen-free water and calibration to provide 1 microgram LPS per gram (g) body weight in 1 mL of diluent. Rats in groups 1 and 3 received iv injections of 1mL pyrogen-free water only.

### *Quantification of Bacterial Endotoxin in Plasma*

An advanced method for optimization of detection of bacterial LPS in plasma with the Limulus test, described by Roth *et al.*[28] was used. All glassware was rendered endotoxin-free by washing with alkaline detergent (E-Toxa-Clean, Sigma), autoclaving for 45 minutes (min), and heating at 190°C for 4 hours (h). Endotoxin-free plasma was obtained from a healthy volunteer. In brief, endotoxin stock solutions (E.coli LPS B, 055:B5) with various concentrations were prepared in pyrogen-free 0.15 mol/liter (L) NaCl (Sigma). 1 volume of each stock solution was added to 9 volumes of endotoxin-free plasma and endotoxin-spiked plasma samples were thus prepared. In order to obtain maximal detection of low concentrations of LPS, plasma samples were diluted fourfold with 0.15 mol/L NaCl followed by heating at

60°C for 30 min. A 0.2 mL sample of plasma (endotoxin-free, endotoxin-spiked, or plasma to be tested) was mixed with 0.6 mL 0.15 mol/L NaCl and incubated in a cork-stoppered tube at 60°C for 30 min. A 0.05 ml sample of this diluted-heated plasma was then incubated with 0.05 mL sample of Limulus lysate (E-Toxate, Sigma) for 30 min in a 37°C water bath. 0.2 mL of chromogenic substrate (S-2423 AB Kabi, Vitrum, Molndal, Sweden) was then added to each assay tube, and incubated for an additional 10 min at 37°C. Reactions were stopped by 0.15 mL of 50% acetic acid, and absorbance was measured at 405 nm in a spectrophotometer. The results were expressed in picrogram (pg) /mL.

### *Liver Histology*

Specimens of liver were fixed in 10% buffered formalin, then processed for histological examination and stained using hematoxylen and eosin (H&E), periodic acid-Schiff (PAS) with diastase digestion, methyl green pyronine, Gomoris reticuline, Masson's trichrome, or phosphothungistic acid hematoxylen (PTAH) in order to assess hepatic abnormalities including necrosis, hydropic degeneration, regenerative activity, decreasing glycogen content of hepatocytes, polymorphonuclear cell (PMNC) infiltration and mononuclear cell infiltration in the portal tracts, pseudoductular proliferation, interlobular duct changes, fibroblastic activity, KC abnormalities, PMNC and, mononuclear cell infiltration in the sinusoids, sinusoidal vascular congestion, portal vascular congestion, sinusoidal vascular thrombosis, portal vascular thrombosis, phlebitis of portal and central veins, and arterial wall changes. Each parameter of each rat was graded by two blinded observers semiquantatively depending on the degree of the abnormality: grade 0-absent, grade 1-mild, grade 2-moderate, and grade 3-severe. A scoring system similar to that of Lichtman and associates[29] was used. The means and standard deviations (SD) were calculated, and the parameters were scored from 0 to 4 for each group according to the corresponding mean values of sham-operated rats. A score of 1 was obtained for example, when the mean value exceeded the mean + 1 SD derived from the sham-operated rats.

### *Bacterial Cultures*

Peritoneal fluid and cardiac blood samples were immediately inoculated on blood agar, EMB agar and prereduced brain-heart infusion (BHI) agar plates. Luminal contents of the jejunal loops or corresponding 10 cm jejunal segments were flushed with 10 mL saline, diluted in thioglycollate broth from $10^{-1}$ to $10^{-10}$, and inoculated on blood agar and BHI plates. Anaerobic culture plates were kept in anaerobic jars (Oxoid) containing gas-generating kit and catalyzer. All specimens were inoculated at 37°C for 72h. Total anaerobic bacterial colony-forming units (CFU) per mL of luminal contents were then determined in order to confirm the development of SBBO in SFBLs within the time limits of this study. Luminal contents were not subcultured to identify the involved anaerobic species.
Gamma-glutamyl transpeptidase (GGT) levels were measured with a Beckman Astra-8 Enzyme Autoanalyzer in serum obtained by centrifugation of the cardiac blood samples at 4000 revolutions per min for 10 min. The results were expressed in U/L.

### *Statistical Analysis*

The results are presented as mean values ±SEM. Mann-Whitnney-U test was used for all comparisons, and a *p* value of less than 0.05 was considered to represent a significant difference. Comparisons of the histological parameters were performed using Kruskal-Wallis test.

### RESULTS

Rats in which complications, such as intraabdominal bleeding, intestinal obstruction, or anastomotic leakage, developed were excluded from the study. All such complications and deaths occurred within the first 72 h postoperatively, and never after. When harvesting laparatomy was performed 14 days later, SFBLs were grossly dilated and were filled with fecaloid material in contrast with SEBLs.

### *Bacterial Counts and Plasma LPS Levels*

Aerobic and anaerobic cultures of peritoneum and blood were negative in all animals. Luminal bacterial concentrations and differential counts, as well as

**Table 1** Plasma LPS and serum GGT levels of the study groups

| | LPS(pg/mL) | GGT (U/L) |
|---|---|---|
| Group 1 SHAM A | 32.10 ± 9.6 | 8.33 ± 1.6 |
| Group 2 SHAM + LPS | - | 8.67 ± 1.5 |
| Group 3 BDL | 142.77 ± 20.5 | 24.86 ± 1.9 |
| Group 4 BDL + LPS | - | 57.50 ± 5.2 |
| Group 5 BDL + SFBL | 352.63 ± 115.3 | 60.29 ± 4.1 |
| Group 6 BDL + SEBL | 86.19 ± 15.7 | 21.67 ± 2.1 |

bacterial cultures of liver, spleen and mesenteric lymph nodes, had been well-documented in previous investigations, and SFBLs had been shown to result in SBBO with 1 week following surgery[26,27,29]. In accordance, rats with SFBLs were shown to have $10^{8-9}$ colony-forming units (CFU) of anaerobic bacteria per mL of the loop content. SEBLs contained approximately $10^{3-4}$ bacteria/mL.

The numerical data regarding plasma LPS and serum GGT levels are shown in Table 1. BDL resulted in significantly elevated plasma LPS concentrations compared with those of the sham-operated group ($p < 0.05$). When BDL was combined with SEBL, plasma LPS levels were similar to those of BDL rats ($p > 0.05$). On the contrary, plasma LPS levels of rats with BDL combined with SFBL were significantly higher than those of BDL rats ($p < 0.05$), indicating that SBBO in animals with BDL resulted in even higher levels of endotoxemia. Groups 2 and 4 were not tested for plasma LPS concentrations because of the existence of recent exogenous LPS administration.

### *Histological and Biochemical Evidence of Liver Injury*

Sham-operated rats (group 1) had random, small mononuclear aggregates, minimal round cell infiltration around bile ducts, and mild congestion affecting both portal and central veins (data not shown). The scores obtained for each involved parameter according to the corresponding mean value of the sham-operated group are given in Table 2. Although simply suggestive of the resultant degree of liver injury (Figure 1), the total scores should be interpreted cautiously because each parameter has a different pathological meaning and deserves special discussion, rather than simple cumulation.

Sham + LPS rats (group 2) had rounded KCs, mild PMNC infiltration in sinusoids, and mild mononuclear cell infiltration in portal tracts and sinusoids. In group 3 (BDL), mild hepatocyte necrosis with eosinophilic degeneration, enlarged portal tracts, pseudoductular proliferation, denser mononuclear infiltration, and portal vascular congestion were noticed (Figure 2). When BDL was combined with iv LPS (group 4), more severe portal and paranchymal alterations were noted (Figure 3). Pseudoductular proliferation was more prominent, and tended to make spurs into the paranchyme. Fibroblastic activity appeared to accompany this proliferation. Inflammation of duct epithelia and portal veins were apparent. KCs appeared more rounded with a tendency to bulge into sinusoids, and they were strongly PAS (+) after diastase digestion. Sinusoidal PMNC and mononuclear cell infiltration was dense. Focal paranchymal necrosis and PMNC were aggregates extensive. Rarely, PTAH (+) eosinophilic material with linear configuration was seen and considered to be fibrin. Central vein inflammation was seen in some rats. Group 5 (BDL + SFBL) also had more severe alterations (Figure 4). Group 6 (BDL + SEBL) had portal alterations predominantly. The inflammation in portal tracts were not as striking as it was in group 5. Mild paranchymal necrosis, PMNC infiltration, KC prominence, sinusoidal inflammation, and occasional platelet thrombosis were seen in most of the rats.

**Table 2** The histological scores obtained for the involved parameters according to the corresponding mean values of the sham-operated group

| PARAMETERS | SHAM+LPS | BDL | BDL+LPS | BDL+SFBL | BDL+SEBL |
|---|---|---|---|---|---|
| necrosis | 0 | 1 | 2 | 3 | 1 |
| hydropic degeneration | 1 | 1 | 2 | 1 | 2 |
| regenerative activity | 0 | 1 | 2 | 2 | 1 |
| decreasing glycogen content | 1 | 1 | 2 | 1 | 1 |
| portal PMNC infiltration | 0 | 1 | 3 | 4 | 1 |
| portal mononuclear cell infiltration | 1 | 2 | 4 | 4 | 2 |
| pseudoductular proliferation | 0 | 1 | 2 | 3 | 1 |
| interlobular duct changes | 0 | 1 | 3 | 4 | 1 |
| fibroblastic activity | 0 | 1 | 2 | 3 | 2 |
| KC abnormalities | 1 | 1 | 3 | 4 | 2 |
| sinusoidal PMNC infiltration | 1 | 0 | 3 | 4 | 1 |
| sinusoidal mononuclear cell infiltration | 1 | 1 | 3 | 4 | 1 |
| sinusoidal congestion | 1 | 1 | 1 | 2 | 1 |
| portal vascular congestion | 0 | 1 | 1 | 2 | 1 |
| sinusoidal thrombosis | 1 | 0 | 1 | 2 | 1 |
| portal vascular thrombosis | 0 | 0 | 0 | 1 | 1 |
| portal-central venous phlebitis | 1 | 1 | 4 | 4 | 1 |
| arterial wall changes | 0 | 0 | 0 | 1 | 0 |
| TOTAL | 9 | 15 | 38 | 49 | 21 |

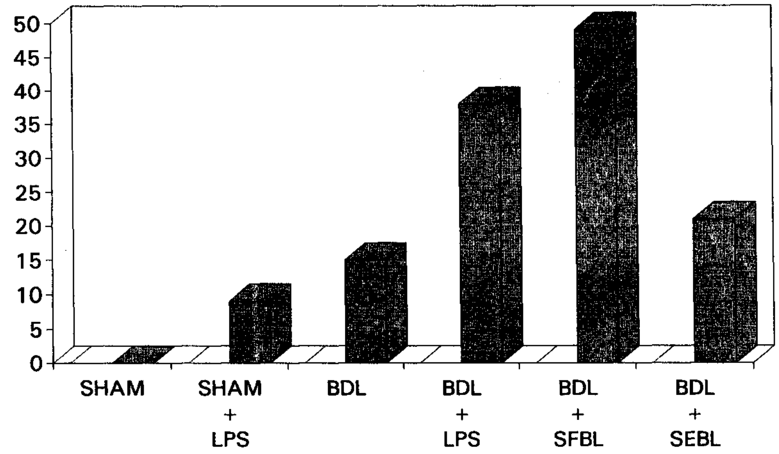

**Figure 1** Total liver injury scores of the study groups (refer to the text).

BDL + SFBL rats had the most striking alterations. In this group the scores of necrosis, portal PMNC and mononuclear infiltration, interlobular duct changes, KC alterations, PMNC and mononuclear cell infiltration in sinusoids, and phlebitis of portal and central veins were found to be significantly higher than those of groups 2, 3 and 6 ($p < 0.05$). Group 4 rats shared these significances for parameters like PMNC and mononuclear cell infiltration in portal tracts, interlobular duct changes, and phlebitis of portal and central veins. Other differences, including those between groups 4 and 5, did not reach statistical significance.

Portal tract alterations predominated in groups 3,4,5 and 6 due to bile duct obstruction. LPS was thought to have a larger impact on paranchymal and sinusoidal components of the liver. Sham + LPS rats

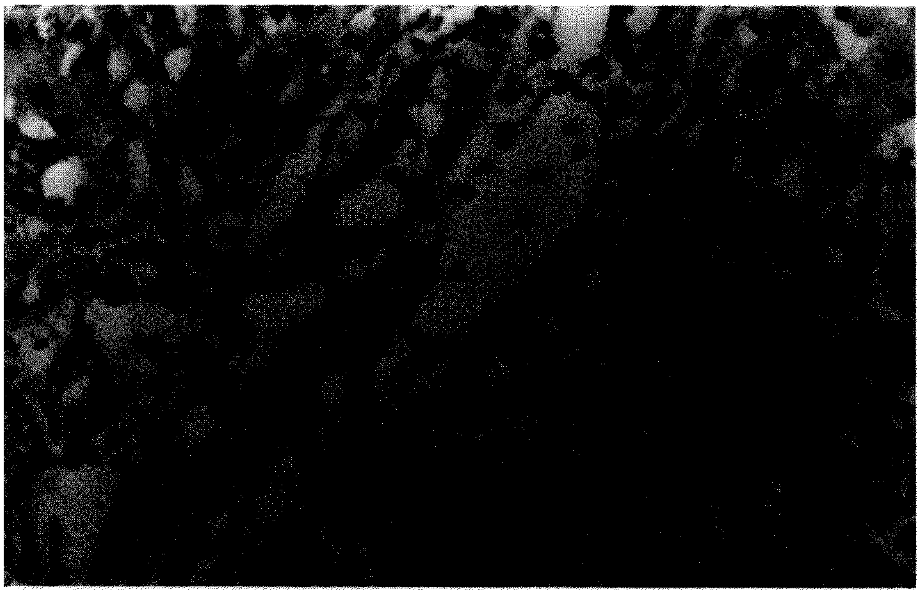

**Figure 2** Histologic section of the liver in group 3 (BDL) (H&E X40 – refer to the text for explanation).

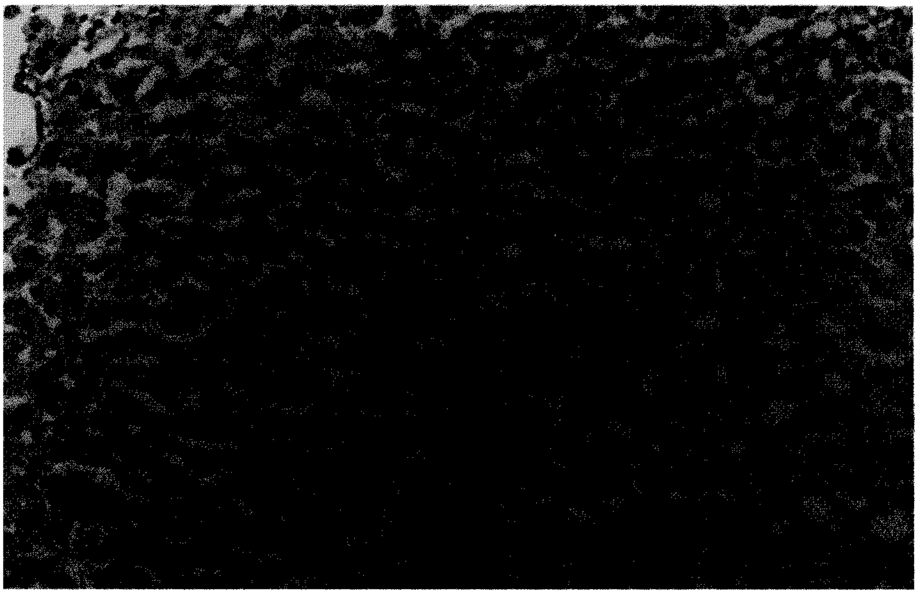

**Figure 3** Histologic section of the liver in group 4 (BDL + LPS) (H&E X 20 – refer to the text for explanation).

had the slightest paranchymal alterations and almost no portal changes. LPS appeared to be more effective on rats with BDL because both portal and paranchymal alterations were more prominent. SEBLs did not produce additional significant changes in rats with BDL.

Serum GGT was not significantly elevated in sham + LPS rats compared with sham-operated controls ($p > 0.05$) (Table 1).BDL caused significantly higher GGT levels compared with those of both sham and sham + LPS groups ($p < 0.05$ for both comparisons). GGT levels were highest in BDL + LPS and BDL + SFBL groups, and differed significantly from all other groups ($p < 0.05$), but not from each other ($p > 0.05$). The mean GGT of rats with BDL + SEBL was not significantly different from that of BDL rats ($p > 0.05$).

### DISCUSSION

".....Given, then, this impressive accumulation of studies in animals and man that suggest a critical role for endotoxins of intestinal origin in a variety of liver injuries and their extrahepatic manifestations, why is this concept so regularly ignored? .....

In part, this neglect may reflect resistance to the notion of mediation... Another factor may be dissatisfaction with present methods to measure and quantitate endotoxins..."

JP Nolan, 1989[4]

A direct cause and effect relationship has been assumed in most studies on the mechanisms of liver injury. As it became clear that endotoxemia may occur and be clinically significant without Gram-negative bacteremia, the concept of gut-originated endotoxemia gained popularity as an important contributor to various disease states[30–32,1,2]. On the basis of these and related studies, it was proposed that: (i) the function of the sinusoidal lining cells of the liver is critical to the viability of the hepatocytes; (ii) that damage to the KCs by a variety of toxic or metabolic injuries leads to impairment of the ability to detoxify endotoxin; (iii) that this primary injury to KCs renders the liver exquisitely sensitive to ordinarily innocuous amounts of gut-derived endotoxins constantly being absorbed; and (iv) the resulting hepatic damage leads to further impairment of LPS detoxification and allows spillover of LPS into the systemic circulation with resulting extrahepatic manifestations[4]. Excess LPS may damage hepatocytes directly[8,9,33]. Moreover KCs, representing terminally differentiated macrophages, release a number of effectors which are critical to the action of LPS and work in concert with LPS and the initiating toxic or metabolic insult at the cellular level. Superoxide and other toxic oxygen radicals, injurious lysosomal enzymes, leukotrienes, as well as a number of potent cytokines, such as tumor necrosis factor (TNF) and platelet activating factor (PAF) are released from recruited macrophages stimulated by LPS[34–37,4,11,12].

The results of the present study provide two important clues that may contribute to our knowledge of

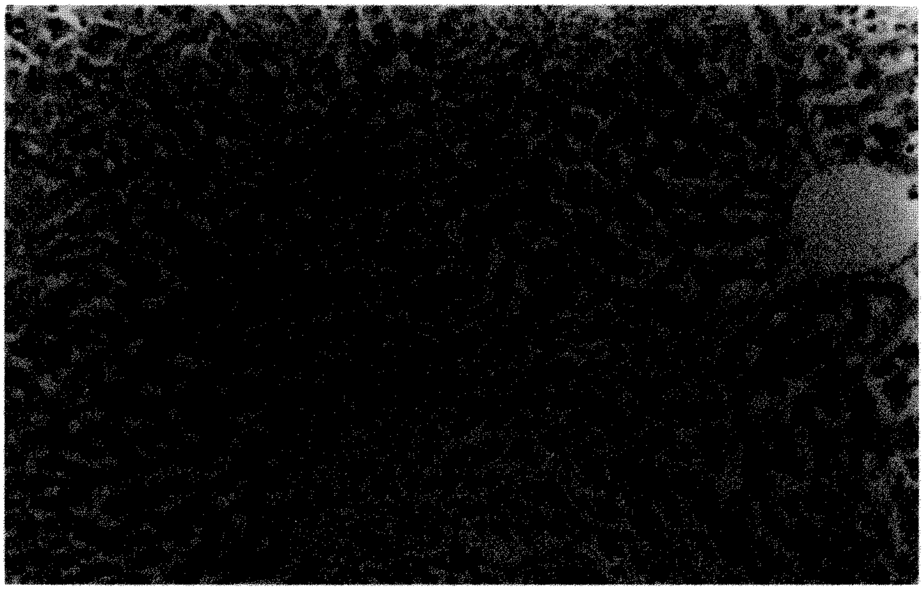

**Figure 4** Histologic section of the liver in group 5 (BDL +SFBL) (H&E X20). Dense inflammation, pseudoductular proliferation tending to make septas into the paranchyme. Sinusoidal inflammation, PMNC aggregation, Kuppfer cell hyperplasia and bulging into the sinusoids, and regenerative activity of the hepatocytes are apparent.

liver injury triggered by extrahepatic bile duct obstruction. First, we can conclude from the histologic and biochemical data that rats with BDL are more susceptible to hepatotoxic effects of even minimal doses of LPS. A more profound liver injury was produced by LPS in those rats with bile duct ligation, suggesting that LPS might have an important role in promoting hepatic damage in extrahepatic bile duct obstruction. Although it was of subordinate importance, the advanced method of plasma endotoxin assay used in this study once again provided experimental evidence of suppression of KC function and spillover of LPS in bile duct obstruction.

After sensitization of the liver to exogenously administered LPS was documented in rats with BDL, the possible contributory role of gut-originated LPS in this model was tested by combining experimental bile duct obstruction with the rat model for SBBO. This method provided us with a perfect model to investigate the role of gut-originated LPS in hepatic injury triggered by BDL.

In this study, a more profound liver injury, confirmed both histologically and biochemically, was produced in rats with BDL + SFBL, in contrast with the control group which consisted of rats with BDL + SEBL. Viable bacteria were not recovered from the blood or peritoneum of rats with SFBLs, so there was no evidence of septicemia or peritonitis in rats with SBBO to account for the worsening of hepatic damage. Plasma LPS levels of all groups correlated well with the histological liver injury, as well as serum GGT levels. The highest circulating levels of LPS were noted in rats with the highest degree of hepatic injury, that is in rats with BDL + SFBL. Plasma LPS levels and liver injury scores of BDL animals did not differ significantly from those of the group with BDL + SEBL. The results, as a whole, suggest that gut-originated endotoxins may be a key factor in promoting the liver injury triggered by extrahepatic bile duct obstruction. We cannot contradict that other bacterial cell wall products, such as peptidoglycan-polysaccharide polymers with well-known inflammatory properties[38–41], might contribute to the resultant hepatic injury in this model.

The liver in extrahepatic bile duct obstruction, we can conclude, is exquisitely sensitive to minor amounts of endotoxin. Consequently, vigorous attempts should be undertaken to prevent or treat any infectious foci in these cases. Another deduction may be that if stagnation of luminal contents and consequent SBBO already exist due to conditions such as partial bowel obstruction, previously created blind loops, small intenstinal diverticula, or motility disorders, a more severe liver injury may result in case of superimposed bile duct obstruction. More important is the suggestion that therapy directed against intestinal endotoxin pool and/ or LPS-mediated effectors might offer a new approach to modify or hamper liver injury in these cases. Further study on the concept of gut-originated LPS mediation of hepatic injury is required to elucidate the responsible mechanisms and the involved LPS-mediated effectors for a better understanding of hepatic injury associated with bile duct obstruction.

### ACKNOWLEDGEMENTS

The study was presented at the 1st European Congress of Word Association of Hepato-Pancreato-Biliary Surgery, 8–11 June 1993, Paris, France.
The study received support for suture materials from Ethicon Limited, International Division, Edinburgh, UK.

### REFERENCES

1. Bhagwandeen, B.S., Apte, M., Manwaring, L., Dickeson, J. (1987) Endotoxin induced hepatic necrosis in rats on an alcohol diet. *Journal of Pathlogy*, **15**, 7–53.
2. van Deventer, S.J.H., ten Cate, J.W., Tytgat, G.N.J. (1988) Intestinal endotoxemia – clinical significance. *Gastoroenterology*, **94**, 825–831.
3. Nolan, J.P., Camara, D.S. (1989) Intestinal endotoxins as cofactors in liver injury. *Immunological Investigation*, **18**(1–4), 325–337.
4. Nolan, J.P. (1989) Intestinal endotoxins as mediators of liver injury – an idea whose time has come again. *Hepatology*, **10**(5), 887–891.
5. Utili, R., Abernathy, C.O., Zimmerman, H.J. (1976) Cholestatic effects of Escherichia coli endotoxin on the isolated perfused rat liver. *Gastroenterology*, **70**, 248–253
6. Ferluga, J., Allison, A.C. (1978) Role of mononuclear infiltrating cells in pathogenesis of hepatitis. *Lancet*, ii 610–611.
7. Bradfield, J.W.B., Wells, M. (1978) Liver disease caused by lysosomal enzymes released from Kupffer cells. *Lancet*, ii 836.
8. McGivney, A., Bradley, S.G. (1979) Action of bacterial endotoxin and lipid A on mitochondrial enzyme activities of cells in culture and subcellular fractions. *Infection and Immunity*, **25** 664–671.
9. Pagani, R., Portoles, M.T., Diza-Laviada, I., Municio, A.M. (1988) Morphological damage induced by E. coli lipopolysaccharide in cultured hepatocytes: Iocalization and binding properties. *British Journal of Experimental Pathology*, **69**, 537–549.
10. Arthur, M.J.P., Kowalski-Saunders, P., Wright, R. (1988) Effect of endotoxin on release of reactive oxygen intermediates by rat hepatic macrophages.
11. Tiegs, G., Wendel, A. (1988) Leukotriene-mediated liver injury. *Biochemical Pharmacology*, **37**, 2569–2573.
12. Thiele, D. (1989) Tumor necrosis factor, the acute phase response and the pathogenesis of alcoholic liver disease. *Hepatology*, **9**, 497–499.
13. Nolan, J.P. (1975) The role of endotoxin in liver injury. *Gastroenterology*, **69**, 1346–1356.
14. Nolan, J.P. (1981) Endotoxin, reticuloendothelial function and liver injury. *Hepatology*, **1**, 458–461.
15. Mills, L.R., Scheuer P.J. (1985) Hepatic sinusoidal macrophages in alcoholic liver disease. *Journal of Pathology*, **147**, 127–132.
16. Cameron, G.R., Hasan, S.M. (1958) Disturbance and structure and function in the liver as the results of biliary obstruction. *J Pathology and Bacteriology*, **75**, 333–349.
17. Dickson, E.R., Grambsch, P.M., Fleming, T.R., Fisher, L.D., Langworthy, A. (1989) Prognosis in primary biliary cirrhosis: model for decision making. *Hepatology*, **10**, 1–7.
18. Wiesner, R.H., Grambsch, P.M., Dickson, E.R., Ludwig, J., MacCarty, R.L., Hunter, E.B., Fleming T.R., Fisher, L.D., Beaver, S.J., LaRusso, N.F. (1989) Primary sclerosing cholangitis: natural history, prognostic factors and survival analysis. *Hepatology*, **10**, 430–436.
19. Pain, J.A., Bailey, M.E. (1987) Measurement of operative plasma endotoxin livels in jaundiced and non-jaundiced patients. *European Surgical Reseach*, **19**, 207–216.
20. Wait, R.B., Kahng, K.U. (1989) Renal failure complicating obstructive jaundice. *American Journal of Surgery*, **157**, 256–263.
21. Holman, J.M. Jr, Rikkers, L.F. (1982) Biliary obstruction and host defense failure. *Journal of Surgical Research*, **32**, 208–213.
22. Katz, S., Grosfeld, J.L., Gross, K., Plager, D.A., Ross, D., Rosenthal, R.S., Hull, M., Weber, T.R. (1984) Impaired bacterial clearance and trapping in obstructive jaundice. *Annals of Surgery*, **199**, 14–20.
23. Pain, J.A., Bailey, M.E. (1986) Experimental and clinical study of lactulose in obstructive jaundice. *British Journal of Surgery*, **73**, 775–778.
24. Nolan. J.P., Leibowitz, A.I. (1978) Endotoxin and the liver III. Modification of acute carbon tetrachloride injury by polymixin B – an antiendotoxin. *Gastroenterology* **75**, 445–449.
25. Cameron, D.G., Watson, G.M., Witts, L.J. (1949) The experimental production of macrocytic anemia by operations on the intestinal tract. *Blood*, **4**, 803–815.
26. King, C.E., Toskes. P.P. (1981) Protein-loosing enteropathy in the human and experimental rat blind loop syndrome. *Gastroenterology*, **80**, 504–509.
27. Lichtman, S.N., Shermann, P., Forstner, G. (1986) Production of secretory lgA in rat self filling blind loops: local slgA immune response to luminal bacterial flora. *Gastroenterology*, **91**, 1495–1502.
28. Roth, R.I., Levin, F.C., Levin, J. (1990) Optimization of detection of bacterial endotoxin in plasma with the Limulus test. *Journal of Laboratory and Clinical Medicine*, **116**, 153–161.
29. Lichtman, S.N., Sartor, R.B., Keku, J., Schwab, J.H. (1990) Hepatic inflammation in rats with experimental small bowel bacterial overgrowth. *Gastroenterology*, **98**, 414–423.
30. Broitman, S.A., Gottlieb, L.S., Zamcheck, M. (1964) Influence of neomycin and ingested endotoxin in the pathogenesis of choline deficiency cirrhosis in the adult rat. *Journal of Experimental Medicine*, **119**, 633–641.
31. Hyland, G., Stein, T., Wise, L. (1977) Abnormalities of liver function following extensive jejunoileal bypass and resection in rats. *Surgery*, **81**, 578–582.
32. Editorial Lancet (1982) Endotoxin and Cirrhosis. *Lancet*, **1**, 318–319.
33. Pagani, R., Portoles, M.T., Bosch, M.A., Diaz-Lavida, I., Municio, A.M. (1987) Direct and mediated Escherichia coli lipopolysaccharide action in primary hepatocyte cultures. *European Journal of Cell Biology*, **43**, 243–246.
34. Arthur, M.J.P., Kowalski-Saunders, P., Wright, R. (1986) Corynobacterium parvum – elicited hepatic macrophages demonstrate enhanced respiratory burst activity compared with resident Kupffer cell in the rat. *Gastroenterology*, **91**, 174–181.
35. Arthur, M.J.P., Kowalski-Saunders, P., Wright, R. (1988) Effect of endotoxin on release of reactive oxygen intermediates by rat hepatic macrophages. *Gastroenterology*, **95**, 1588–1594.
36. Beutler, B., Cerami, A. (1987) Cachectin: more than a tumor necrosis factor. *New England Journal Of Medicine*, **316**, 379–385.
37. Caramelo, C., Fernandez-Gallardo, S., Santos, J.C., Inarrea, P., Sanchez Crespo, M., Lopez-Novoa, J.M., Hernado, L. (1987) Increased levels of platelet activating factor in blood from patients with cirrhosis of the liver. *European Journal of Clinical Investigation*, **17**, 7–11.
38. Stimpson, S.A., Schwab, J.H., Janusz, M.J., Anderle, S.K., Brown, R.R., Cromartie, W.J. (1986) Acute and chronic inflammation induced by peptidoglycan structures and polysaccharide complexes. In Biological Properties of Peptidoglycan, edited by PH Seidl and KH Schleifer, pp. 273–290. Berlin: de Gruyter
39. Sartor, R.B., Cromartie, W.J., Powell, D.W., Schwab, J.H. (1985) Granulamatous enterocolitis induced in rats by purified bacterial cell wall fragments. *Gastroenterology*, **89**, 587–595.
40. Wahl, S.M., Hunt, D.A., Allen, J.B., Wilder, R.L., Paglia, L., Hand, A.R. (1986) Bacterial cell wall induced hepatic granulomas. *Journal of Experimental Medicine*, **163**, 884–902.
41. Lichtman, S.N., Keku, J., Schwab, J.H., Sartor, R.B. (1991) Hepatic injury associated with small bowel bacterial overgrowth in rats is prevented by metronidazole and tetracycline. *Gastroenterology*, **100**, 513–519.

